# Experience of introducing an electronic health records station in an objective structured clinical examination to evaluate medical students’ communication skills in Canada: a descriptive study

**DOI:** 10.3352/jeehp.2023.20.22

**Published:** 2023-07-04

**Authors:** Kuan-chin Jean Chen, Ilona Bartman, Debra Pugh, David Topps, Isabelle Desjardins, Melissa Forgie, Douglas Archibald

**Affiliations:** 1 Department of Emergency Medicine, Faculty of Medicine, University of Ottawa, Ottawa, ON, Canada; 2 Medical Council of Canada, Ottawa, ON, Canada; 3 Department of Medicine, Faculty of Medicine, University of Ottawa, Ottawa, ON, Canada; 4 Department of Family Medicine, Cumming School of Medicine, University of Calgary, Calgary, AB, Canada; 5 Department of Family Medicine, Department of Innovation in Medical Education, Faculty of Medicine, University of Ottawa, Ottawa, ON, Canada; Hallym University, Korea

**Keywords:** Counseling, Feasibility studies, Focus groups, Medical students, Psychometrics

## Abstract

**Purpose:**

There is limited literature related to the assessment of electronic medical record (EMR)-related competencies. To address this gap, this study explored the feasibility of an EMR objective structured clinical examination (OSCE) station to evaluate medical students’ communication skills by psychometric analyses and standardized patients’ (SPs) perspectives on EMR use in an OSCE.

**Methods:**

An OSCE station that incorporated the use of an EMR was developed and pilot-tested in March 2020. Students’ communication skills were assessed by SPs and physician examiners. Students’ scores were compared between the EMR station and 9 other stations. A psychometric analysis, including item total correlation, was done. SPs participated in a post-OSCE focus group to discuss their perception of EMRs’ effect on communication.

**Results:**

Ninety-nine 3rd-year medical students participated in a 10-station OSCE that included the use of the EMR station. The EMR station had an acceptable item total correlation (0.217). Students who leveraged graphical displays in counseling received higher OSCE station scores from the SPs (P=0.041). The thematic analysis of SPs’ perceptions of students’ EMR use from the focus group revealed the following domains of themes: technology, communication, case design, ownership of health information, and timing of EMR usage.

**Conclusion:**

This study demonstrated the feasibility of incorporating EMR in assessing learner communication skills in an OSCE. The EMR station had acceptable psychometric characteristics. Some medical students were able to efficiently use the EMRs as an aid in patient counseling. Teaching students how to be patient-centered even in the presence of technology may promote engagement.

## Graphical abstract

[Fig f4-jeehp-20-22]

## Introduction

### Background/rationale

While studies have found that electronic medical records (EMRs) have potential to improve quality of care [1], there may also be unintended consequences when they are introduced. A systematic review by Alkureishi et al. [2] including 53 studies found that patient-doctor communication could be impacted both positively (e.g., facilitating questions) and negatively (e.g., interrupted speech and low rates of screen sharing). This complex effect was also noted in a scoping review by Crampton et al. [3], in which EMR use was found to impact eye contact, information sharing, and relationship building. There have been some EMR/electronic health record training, pilots, and evaluative studies done in Canada that focus on the learners’ technical skills in using health information technology. However, there is little published literature that describes the assessment of trainees’ EMR-related competencies and none that assess the impact of EMR use on perceived communication skills from the standardized patients’ (SPs) perspectives in Canada [2,4,5].

### Objectives

To address this gap in the literature and to guide the development of assessment tools, this study explored the impact of EMR use on communication skills. This paper presents a novel objective structured clinical examination (OSCE) station that required students to utilize an EMR. The station incorporated a new SP tool that supplemented physician-examiner scoring for the assessment of students’ communication skills in the context of an EMR. SPs’ perceptions of EMR-related communication skills were explored. The specific objectives were as follows: first, to compare students’ performance and item-total correlation in EMR OSCE station with those in other 9 stations; second, to explore the correlation between SPs’ and physician examiners’ evaluations of students’ EMR OSCE performance; third, to compare the SPs’ and physician examiners’ total score between students who used graphs during counseling and those who did not; and fourth, to hear SPs’ perspectives on EMR use in OSCE.

## Methods

### Ethics statement

Ethical approval for this study was granted by the Ottawa Health Science Network Research Ethics Board, the Ottawa Hospital Research Institute, and the University of Ottawa (OHSN-REB ID 20190782-01H). The requirement to obtain informed consent for utilizing de-identified student scores was waived by the research ethics boards. Informed consent was obtained from focus group participants.

### Study design

This descriptive study was conducted to determine the feasibility of introducing an EMR station in an OSCE for evaluating students’ communication. Thematic analysis was also done for the SPs’ perspectives on EMR use in an OSCE.

### Setting

**Fig. 1.**
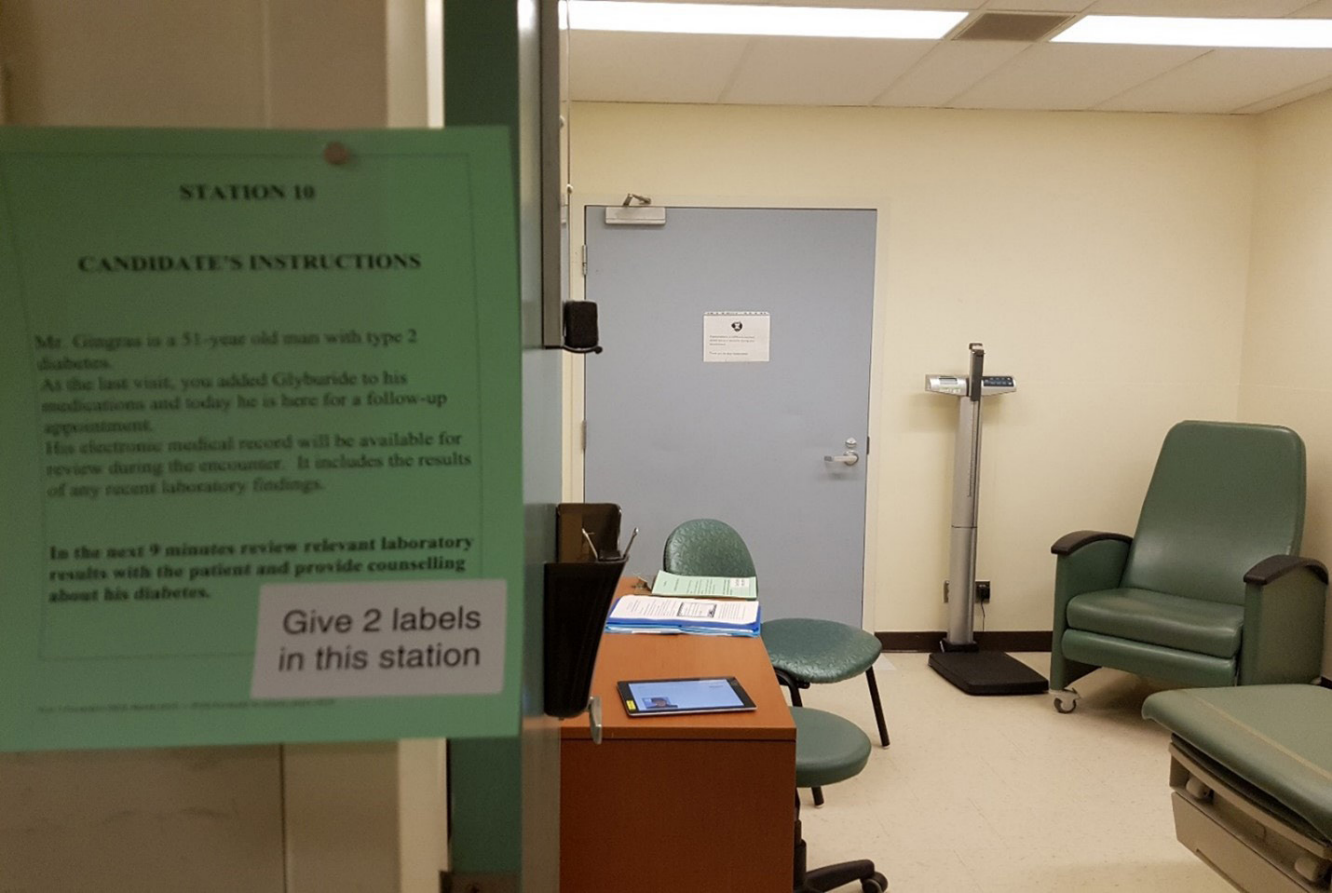
Electronic medical record (EMR) objective structured clinical examination (OSCE) station set up. The iPad on the table has the OpenLabyrinth software with simulated EMR screens for the students to use as part of their diabetes counseling OSCE.

**Fig. 2.**
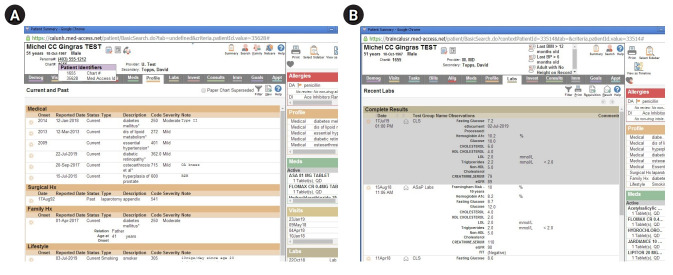
(A, B) Screenshots of the simulated electronic medical record.

**Fig. 3.**
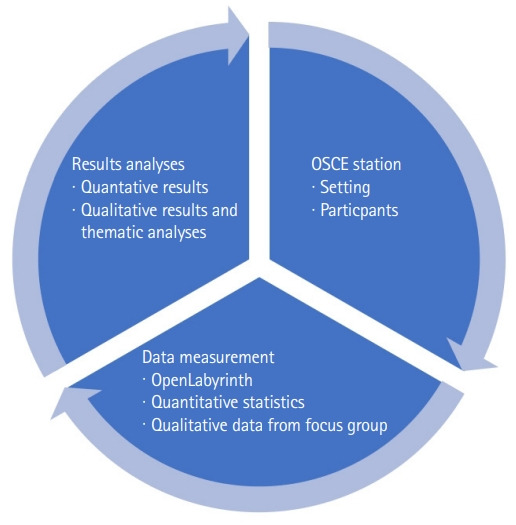
Research and assessment design procedure. OSCE, objective structured clinical examination.

An OSCE station that incorporated a simulated EMR was developed on OpenLabyrinth (https://openlabyrinth.ca/), an open-source education research platform that was able to record the number of times students clicked on the EMR and the duration of time the students stayed on a particular page of the EMR. Students were tasked with counseling an SP with type 2 diabetes after obtaining a relevant history and reviewing their EMR. The students were given 1 minute to read the prompt/instructions posted outside of the room (Fig. 1), where the SP presented to the student’s follow-up clinic appointment to review lab results and to provide further lifestyle counseling about his diabetes. Upon entering the examination room, the student had 7 minutes to review relevant laboratory findings in the EMR and to provide counseling about the SP’s diabetes control. The EMR included relevant information, such as laboratory results and graphs demonstrating elevated hemoglobin A1C (HbA1C) and weight, as well as information that was not germane to the case, requiring students to counsel the patient (Fig. 2). Students could use the EMR as much or as little as they wanted during the OSCE encounter. Students were provided with a 20-minute EMR orientation and training session prior to the start of the first OSCE station. All anglophone 3rd-year medical students were required to participate in this formative OSCE. The research and assessment procedures are presented in Fig. 3. A photograph of the OSCE station is presented in Fig. 2. Screenshots of the simulated EMR are presented in Fig. 3.

### Participants

This pilot station was administered within a 10-track formative OSCE for 99 third-year medical students on March 2, 2020 (before the coronavirus disease 2019 pandemic state of emergency was declared in Ontario on March 17, 2020). There were a total of 10 physician examiners for the 10 stations, and 7 SPs for the EMR pilot station. Three SPs consented to being part of the qualitative study.

### Data variables

The data variables extracted from OpenLabyrinth included the number of EMR pages visited (including if a student returned to a page that was already visited), the total time taken for the case, and the time spent per EMR page. The assessment scores by SPs and physician examiners were used as variables in the comparative analysis.

### Data sources/measurement

OpenLabyrinth was used to track the students’ use of the EMR, including access to graphs, should the students choose to use them during the station. Students were assessed by 10 physician examiners using a station-specific checklist (Supplement 1) and rating scales. In addition, 7 SPs completed rating scales related to communication (Supplement 2) [6]. Medical students’ responses and raters’ records were available from Dataset 1. Three SPs consented to the qualitative portion of the study; they reconvened with 3 of the investigators (K.J.C., D.A., I.B.) for a focus group 1 week after the OSCE. During the focus group discussion, the 3 SP participants were asked to discuss their perspective on the use of EMRs when interacting with the OSCE candidates. OSCE scores and focus group qualitative data from interviews with 3 SPs were de-identified before analysis.

### Statistical methods

Station descriptive statistics and item total correlations (ITCs) were calculated. The students’ performance scored by physician examiners on the EMR pilot station was compared to the other 9 OSCE stations using descriptive statistics and ITCs. The content of other OSCEs included patient education and counseling, diagnosis, physical exam, and history. ITCs were used to test if student performance on the EMR station varied in line with other OSCE stations. Correlations between SP and physician examiner scoring were assessed via Spearman rank correlation coefficients.

Finally, the independent group t-test was used to compare the SPs’ and physician examiners’ scores between the students who used graphs during counseling (N=84 for HbA1C and N=53 for weight) and those who did not (N=15 for HbA1C and N=46 for weight). Student allocation was not randomized. Focus group data were analyzed using thematic analysis (Supplement 3) [7]. Whole transcripts are available from Supplement 4. Three investigators (K.J.C., D.A., I.B.) independently analyzed the transcript to inductively develop preliminary codes and negotiated discrepancies to establish consensus. The data were then interpreted to develop themes and subthemes [7]. OSCE scores and focus group data were de-identified before analysis.

## Results

### Main results

Comparison of students’ performance and ITC at the EMR OSCE station with those at the other 9 stations. Compared to the other 9 stations in this OSCE, the mean score at the EMR station was one of the lowest (5.55); the pilot EMR station also had the lowest minimum score (1.2) and the lowest pass rate (68%), with an ITC of 0.217, indicating that the station discriminated well between strong and weak examinees (Supplement 5).

### Correlation between SP and physician examiners’ evaluations of students’ performance on the EMR OSCE station

The relationship between SP and physician examiner scoring was examined via Spearman correlation coefficients. The Spearman correlation coefficient is defined as the Pearson correlation coefficient using rank variables. Spearman’s correlation is a measure of association for ordinal variables such as scales (Table 1).

Given that SPs and physician examiners used different ranking scales (6 points for physician examiners and 5 points for SPs), we collapsed the 2 middle physician examiner rankings (borderline satisfactory and borderline unsatisfactory) into 1 (borderline). The middle ranking for the SP was “adequate.” In a consultation with a subject matter expert, it was decided that collapsing the 2 physician examiner borderline categories would be the closest to the adequate category on the SP rating scale. Consequently, a ranking of 0 on the physician examiner scale was recoded as 1, a ranking of 1 was recoded as 2, rankings of 2 and 3 were coded as 3, a ranking of 4 remained 4, and a ranking of 5 remained 5. There was no correlation between the time a student spent on the EMR and the total SP score.

### Students’ use of graphs and raters’ scores

Use of the HbA1C graphical presentation was not found to be a predictor of the physician examiner station score. The comparison between groups who did and did not use HbA1C graphical presentation was not significant (t[97]=-1.489, P=0.140), indicating there was no difference in the mean physician examiner station score between students who used the HbA1C graphical presentation and those who did not.

Use of the HbA1C graphical presentation was found to be a predictor of the total SP score. The comparison between groups who did and did not use HbA1C graphical presentation was significant (t[97]=-2.079, P=0.041), indicating there was a difference in the mean total SP score between students who used the HbA1C graphical presentation and those who did not. The t-tests indicated that whether or not a student utilized the weight graph had no impact on either the physician examiner station score (t[97]=–0.262, P=0.794) or the total SP score (t[97]=–0.262, P=0.794).

### SPs’ perspectives on EMR use in the OSCE

The focus group revealed 5 main themes surrounding their perception of EMR use’s effect on clinicians’ performance and the OSCE station: technology, communication competency, case design, ownership of health information, and charting and timing of EMR usage (Supplement 6). SPs perceived many of the students’ communication techniques while using EMRs as effective, such as eye contact and integrating data retrieval into conversations. How students used the technology varied greatly, but all SPs commented on the importance of core communication skills, regardless of technology. Training on how to communicate with patients while using the EMR was recommended by all participants. For some SPs, technology was felt to be a distraction, and for others, it was not an issue. Nonverbal communication was highlighted: when students accessed computers, they often failed to maintain eye contact and speech pattern, which are nonverbal communication cues associated with positive affect. SPs viewed the EMR as another means of gaining health information when used by strong communicators; by reviewing graphs together with the student, they were able to actively participate in their care.

In our thematic analysis, focus group data revealed another domain: case design (Supplement 7). In this thematic domain, the SPs commented on both the difficulty of this case and the difficulty of their task in assessing the students. The simulated case was felt to be challenging for both the learners and SPs, due to technological constraints such as the speed of the wireless internet connection, and challenges in navigating their duality of roles in assessing the learners. While additional training was provided to the SPs on the use of their assessment tools, SPs raised their discomfort in being co-examiners with the physician examiners. SPs highlighted that some students voluntarily shared their screen, while others only did so when prompted. SPs expressed decreased rapport when screens were not readily shown and perceived those students as weaker communicators when they were more guarded with the tablet and did not utilize the EMR to provide counseling.

## Discussion

### Interpretation

Our study set out to specifically explore if an EMR station in an OSCE setting is feasible to evaluate students’ communication skills. Unsurprisingly, given the complexity of integrating electronic data, the pilot station had the lowest minimum score and pass rate and one of the lowest mean scores of the 10-station OSCE, suggesting that the pilot station was one of the more difficult stations on this exam. This may be because the performance of patient education in this station involved 3 domains: cognition, skill, and affect. Students needed to demonstrate knowledge underpinning health promotion and recognize various deficits in the SPs’ diabetes control, to display communication skills and competency in using health information technology, and to demonstrate appropriate affect and compassion.

In this study, graphical trends in HbA1C and weight over time were available for the students to use in counseling a simulated patient with type 2 diabetes. Capitalizing on tools available in an EMR, such as the graphical display of laboratory information, can aid patients’ understanding of their health issues and facilitate shared decision-making [8-10]. Use of the HbA1C graph was correlated with the total SP score. Our findings suggest that medical students may be able to leverage the EMR’s functionalities of multimodal information presentation using graphs to counsel patients. In contrast, there was no difference in performance between students who used the weight graph and those who did not. Perhaps this is because changes in weight may be easier to communicate than HbA1C without the visual display of quantitative information.

The correlations provided in Table 1 indicate that the SP EMR utilization rating scale had a significant correlation with physician examiners’ scores for questioning skills and information-giving scales. The physician examiner information-giving rating awarded by the physician examiners was higher when EMR utilization was also scored higher by the SP, indicating that utilizing the EMR enhanced information-giving.

Overall, the pilot station performed well and discriminated well among students, with the novel SP tool evaluated as having acceptable psychometric characteristics. This built upon pre-existing literature suggesting that SPs could reliably discriminate between weak and strong communicators, and by extension, judge the students’ overall performance in some domains [6,11-13]. Furthermore, students’ ability to use the EMR for adherence optimization, measured from the physician examiners’ perspective, correlated with communication skills measured by SPs.

While research suggests that clinicians’ use of EMRs during an encounter can influence communication, such as by leading to decreased focus and increased disruption in conversational sequence [14], our participants expressed that strong baseline communication-related skills are foundational in students’ adaptation to technology (Supplement 7). Students who appeared comfortable with technology were deemed to be stronger communicators, which corresponds to a previous study that indicated that clinicians with stronger computer skills were more likely to reap the communicative benefits of the EMR [15]. Lastly, focus group participants expressed appreciation of students’ use of EMR in health promotion, including the use of graphs. This sentiment echoed past research findings that EMR use can improve patient involvement in decision-making [14].

### Limitations

While the quantitative analyses are encouraging, this is only 1 OSCE station in 1 group of learners. While OpenLabyrinth recorded students’ access to graphs, video recording was not used. As such, a student could have clicked on a graph and then clicked away without using it. The SPs observed students’ use of the graphs more globally and organically. Qualitative analyses were limited to interviewing 3 SPs, who may have variable responses to interview questions depending on personal interest and may be affected by other participants’ responses. This was not a randomized controlled study; thus, it would not be appropriate to draw definitive conclusions regarding the EMR’s influence or effect on communication skills of the medical students. SP perception of EMRs’ effect on communication is a field of potential future research.

### Generalizability

The case was created with transferability in mind, where health promotion and lifestyle modification counseling are typically performed during both primary care and specialty visits. Given the wide adoption of EMRs in both inpatient and outpatient medicine, the findings would be expected to extrapolate to both hospital and clinic-based settings.

### Conclusion

With the increased use of EMRs in Canadian hospitals, it is essential for medical educators to continue to develop cases and assessment tools that address patient-centered communication.

## Figures and Tables

**Figure f4-jeehp-20-22:**
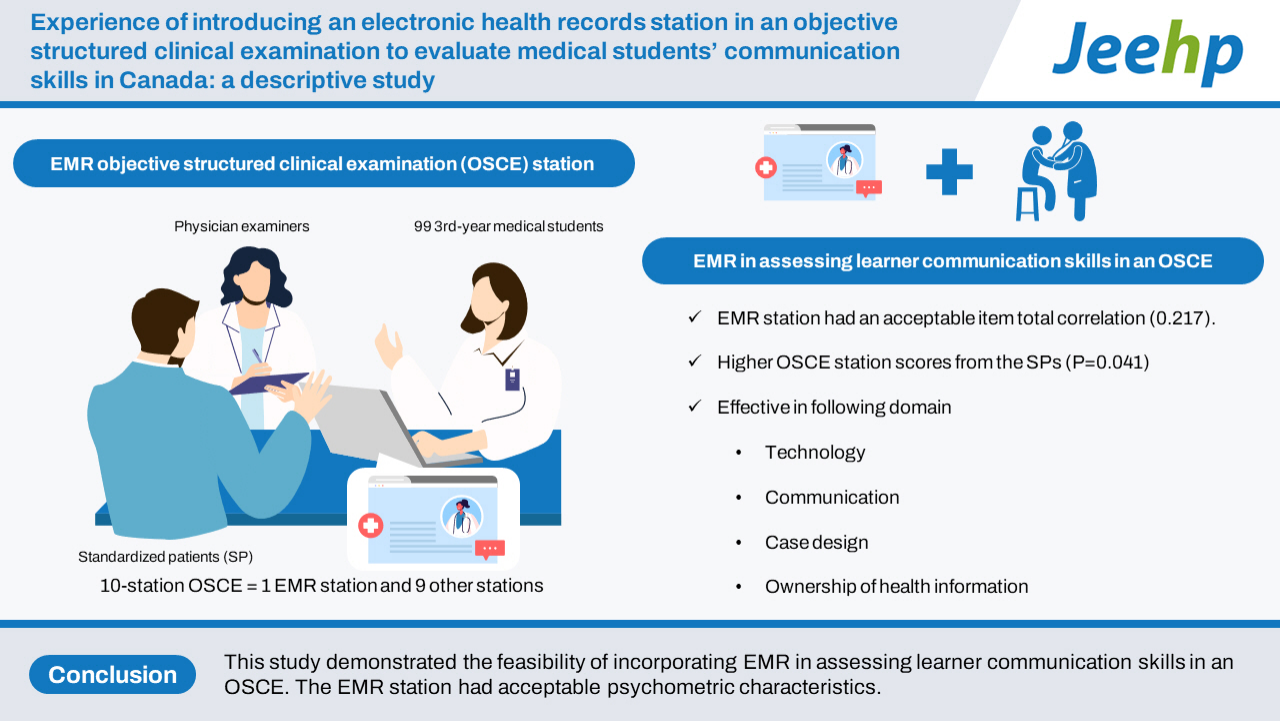


**Table 1.** Correlations between SP and PE scoring on pilot rating scales (Spearman rho correlations)

	SP listening & attentiveness	SP communication	SP empathy & rapport	SP EMR utilization
PE listening skills	0.175	0.145	0.159	**-0.015**
PE questioning skills	0.251^a)^	0.345^b)^	0.202^a)^	0.241^a)^
PE interview organization	0.386^b)^	0.343^b)^	0.318^b)^	0.177
PE information giving	0.345^b)^	0.386^b)^	0.249^a)^	0.224^a)^
PE empathy	0.182	0.139	0.228^a)^	**0.038**
PE respect	0.225^a)^	0.204^a)^	0.250^a)^	0.163
PE adherence optimization	0.340^b)^	0.335^b)^	0.216^a)^	0.18

Statistically significant results are marked in bold. SP, standardized patient; PE, physician examiner; EMR, electronic medical record.

a) Correlation was significant at the 0.05 level (2-tailed).

b) Correlation was significant at the 0.01 level (2-tailed).

## Footnotes

**Data availability**
Data files are available from Harvard Dataverse: https://doi.org/10.7910/DVN/IFMDCC Dataset 1. OpenLabyrinth raw data.

## Supplementary materials

Supplementary files are available from Harvard Dataverse: https://doi.org/10.7910/DVN/IFMDCC

Supplement 1. Physician examiners’ rating scales and their descriptions.

Supplement 2. Standardized patients’ rating scales and their descriptions.

Supplement 3. Iterative steps taken to generate thematic analysis (from Braun V, Clarke V. One size fits all?: what counts as quality practice in (reflexive) thematic analysis? Qual Res Psychol 2021;18:328-352. https://doi.org/10.1080/14780887.2020.1769238).

Supplement 4. Content of focus group transcription.

Supplement 5. Electronic medical record station mean score, pass rate and ITC comparison with other stations.

Supplement 6. Mapping standardized patient rating scales to physician examiner rating scales.

Supplement 7. Focus group data domains, examples, and illustrative quotes.

Supplement 8. Audio recording of the abstract.
